# Supplementary material for: Biomimetic CO oxidation below −100 °C by a nitrate-containing metal-free microporous system
Source: Nat Commun. 2021 Oct 15;12:6033. doi: 10.1038/s41467-021-26157-3 (PMC8519918; doi:10.1038/s41467-021-26157-3)
Supplement: Supplementary file 1 — Supplementary Information [file 41467_2021_26157_MOESM1_ESM.pdf]

Supplementary Information

Biomimetic CO oxidation below -100 ^{0}C by a nitrate-containing metal-free microporous system

Konstantin Khivantsev,[1 †*] Nicholas R. Jaegers,[1†] Hristiyan A. Aleksandrov,[2, †*] Libor Kovarik,[1] Miroslaw A. Derewinski,[1,3] Yong Wang,[1,4] Georgi N. Vayssilov,[2] and Janos Szanyi[1†*]

[1] Institute for Integrated Catalysis, Pacific Northwest National Laboratory, Richland, WA 99352, USA.

[2] Faculty of Chemistry and Pharmacy, University of Sofia, Sofia 1126, Bulgaria

[3]Jerzy Haber Institute of Catalysis and Surface Chemistry, Polish Academy of Sciences, Krakow 30-239, Poland

[4]Voiland School of Chemical Engineering and Bioengineering Washington State University, Pullman, WA, USA99163

* Correspondence to: K.K. E-mail: Konstantin.Khivantsev@pnnl.gov . H.A.A. E-mail: Haa@chem.uni-sofia.bg. J.Sz. E-mail: Janos.Szanyi@pnnl.gov;

[†] These authors contributed equally

Supplementary Figures

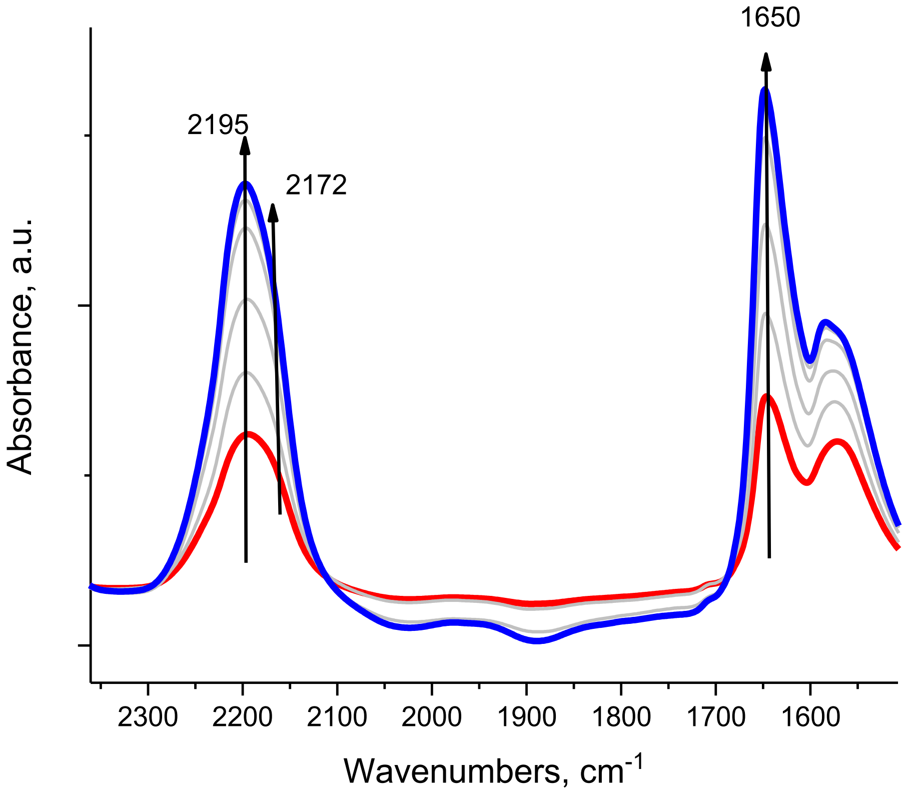

Supplementary Figure 1. Difference spectra during *in-situ* FTIR during sequential NO_2 adsorption (2 Torr) at room temperature on H-SSZ-13 with Si/Al ~ 6.

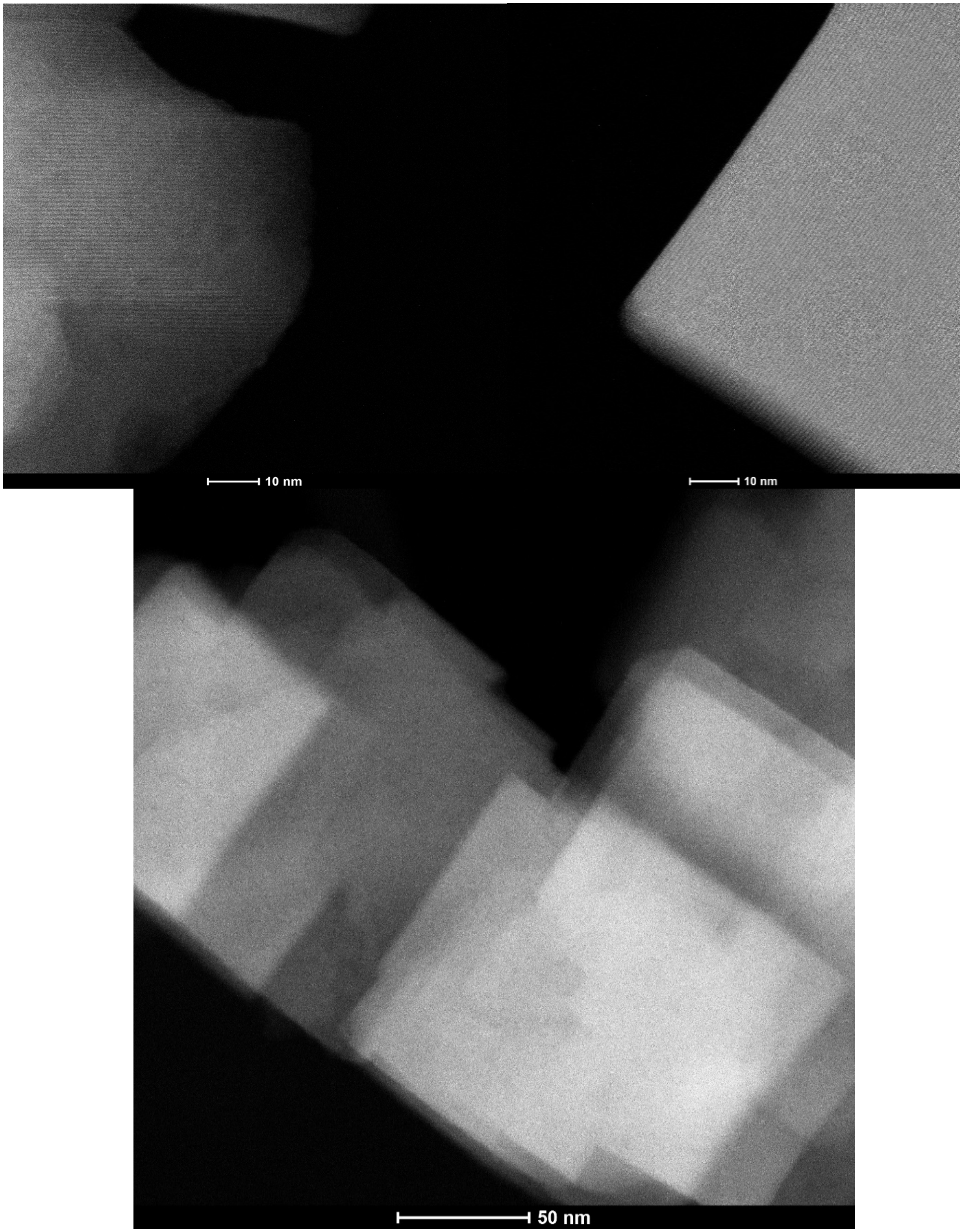

Supplementary Figure 2. Representative HAADF-STEM images of the small-pore SSZ-13 crystals employed in this study, showing highly crystalline materials.

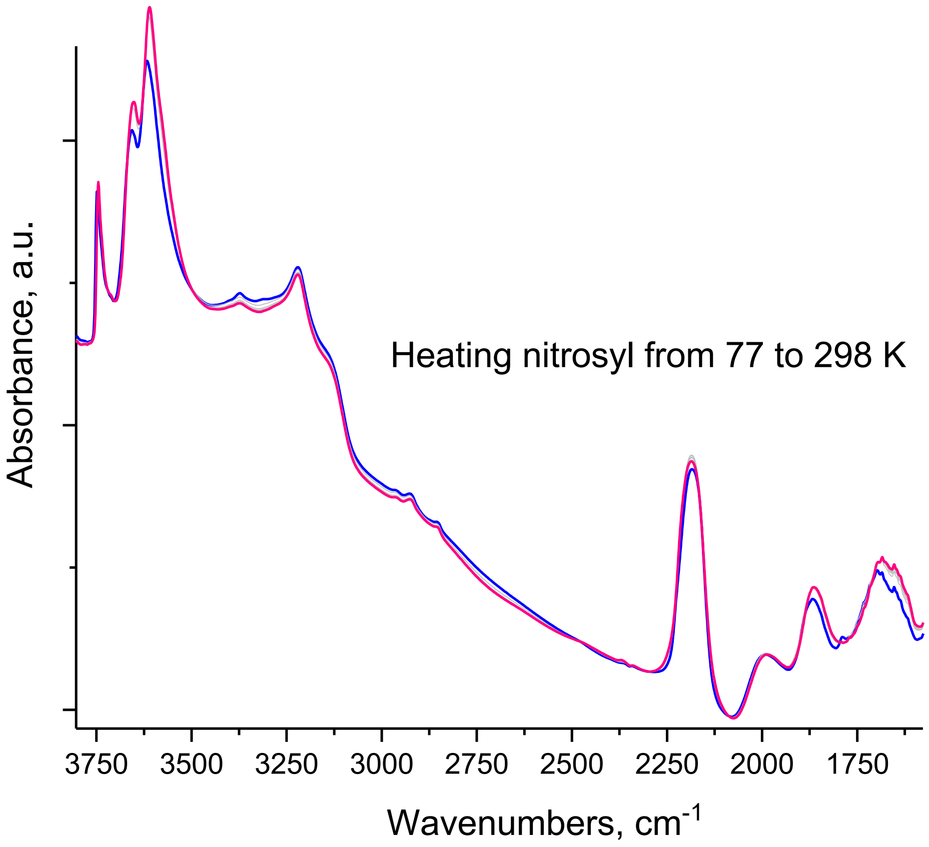

Supplementary Figure 3. In-situ FTIR during heating of nitrosyl (NO^+)/SSZ-13 (Si/Al ~12) from 77 K (blue spectrum) to 298 K (red spectrum). Note that the IR cell vacuum was used as background (not zeolite itself)

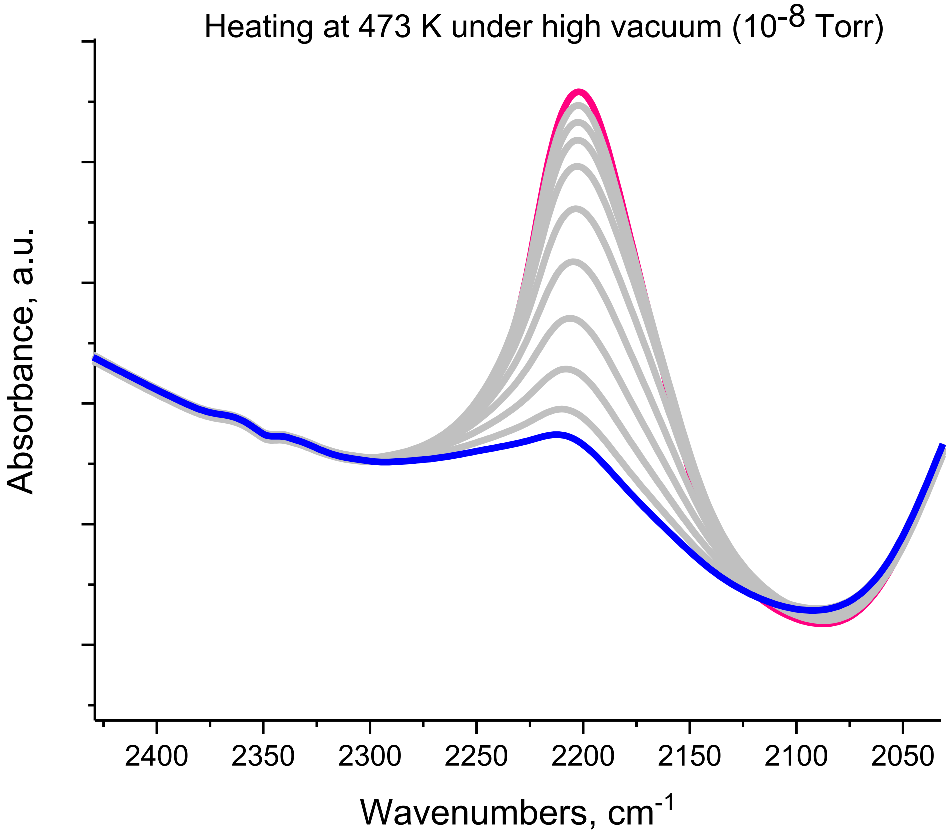

Supplementary Figure 4. In-situ FTIR of the N-O stretching region during continuous heating of Nitrosyl/SSZ-13 (Si/Al ~12) at 200 °C under high vacuum (10^{-8} Torr) from time=0 (red spectrum) to time=1 hour (blue spectrum). The intermediate spectra are shown in gray.

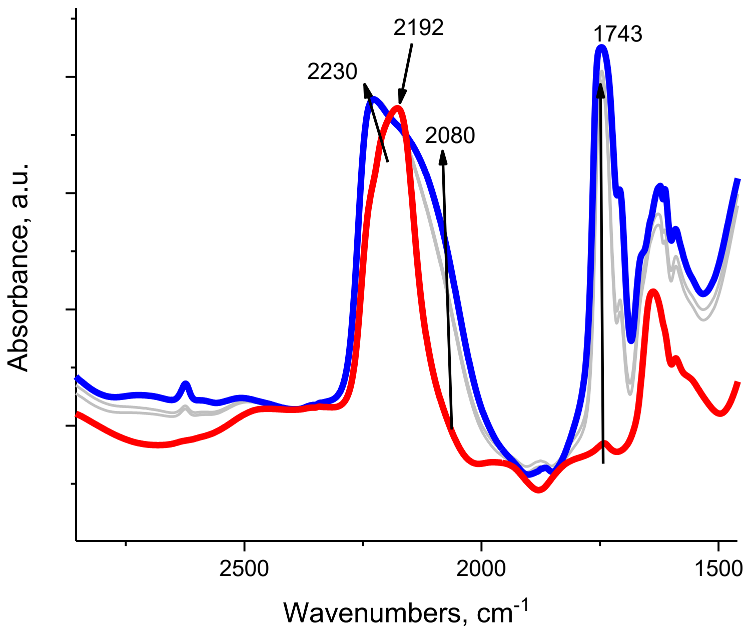

Supplementary Figure 5. FTIR during in-situ adsorption of excess NO_2 (2 Torr) on NO^+/SSZ-13 (Si/Al ~6) obtained through NO_2 disproportionation. The initial NO^+/SSZ-13 spectrum is shown in red. The intermediate spectrum is shown in gray. The final state after 2 Torr NO_2 adsorption is shown in blue.

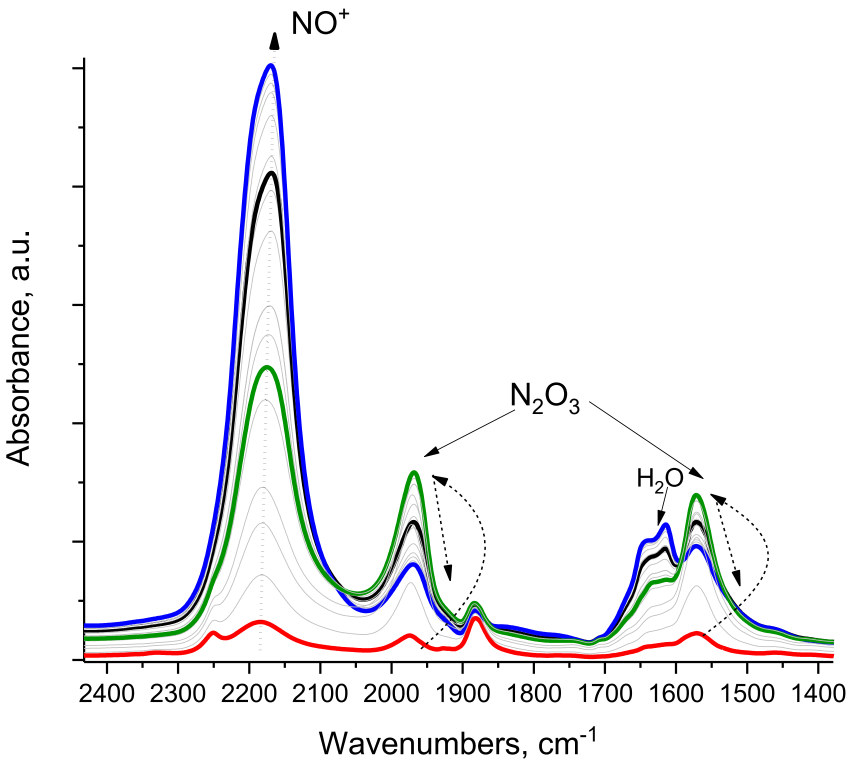

Supplementary Figure 6. In-situ FTIR during NO (0.5 Torr) and O_2 (0.1 Torr) co-adsorption on H-SSZ-13 (Si/Al~12). The red spectrum is after the first pulse of (NO+O_2). The blue spectrum is at the end of the measurement (~ 15 minutes). The gray spectra were recorded approximately through equal time intervals between the start and the end of the measurement. The green (intermediate) spectrum is highlighted because it shows that N_2O_3 that is formed from (NO+NO_2) interaction at this point reaches its maximum concentration: as it gets formed, it gets consumed by Bronsted acid protons forming NO^+ and H_2O as clarified in the main text (the black spectrum shows one of the snapshots of this consumption resulting in loss of N_2O_3 spectroscopic signatures, growth of NO^+ and H_2O features). For spectra collection, H-SSZ-13 zeolite pellet was used as a background.

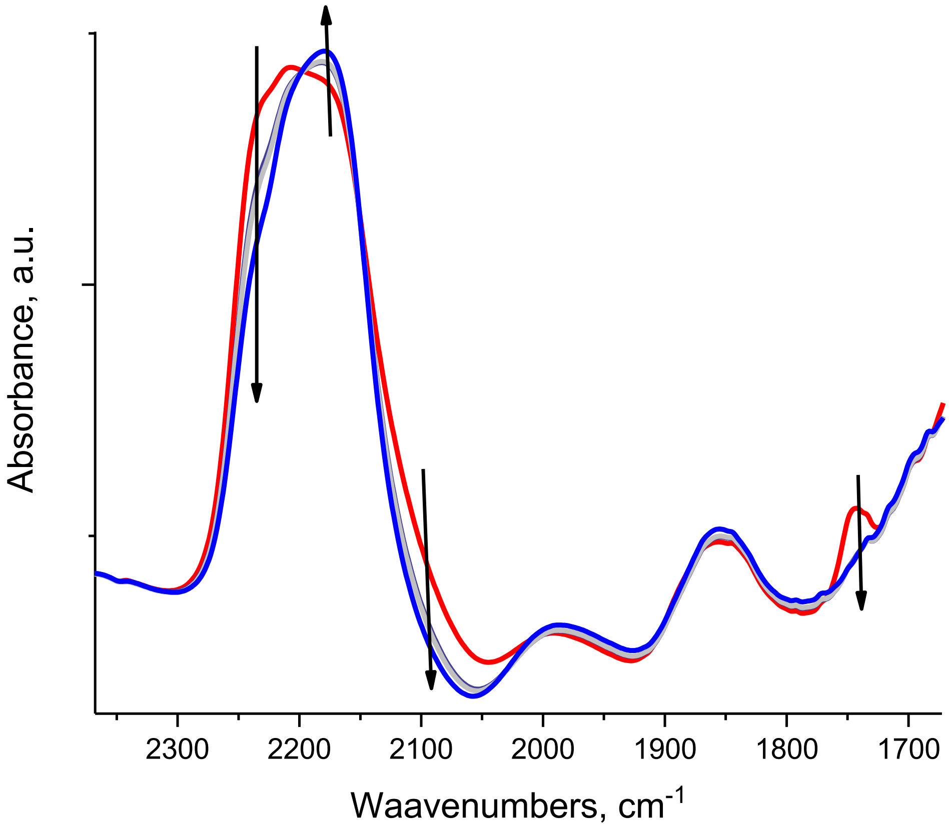

Supplementary Figure 7. In-situ FTIR during desorption of excess NO_2 (2 Torr) from NO^+-NO_2 complex hosted in SSZ-13 (red initial spectrum) under continuous vacuum (10^{-7} Torr) for 10 minutes for H-SSZ-13 (Si/Al ~12). Intermediate spectra are denoted in gray. The final spectrum is shown in blue. Data are complementary to Figure 1C.

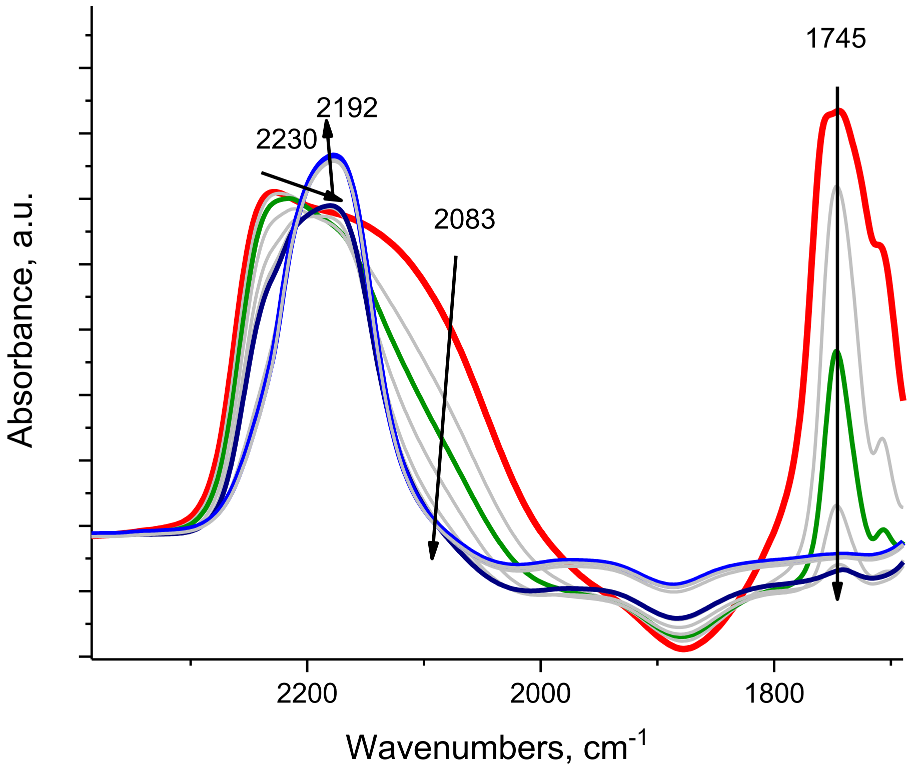

Supplementary Figure 8. In-situ FTIR during desorption of excess NO_2 (2 Torr) from NO^+-NO_2 system under continuous vacuum (10^{-7} Torr) for ~15 minutes for H-SSZ-13 (Si/Al ~ 6) at 298 K. The initial spectrum is denoted in red. The final spectrum is denoted in blue. Intermediate spectra taken in approximately equal time intervals are denoted in gray (with two intermediate spectra denoted in green and black to show the evolution and correlations of different N-O stretches). For spectra collection, H-SSZ-13 zeolite pellet was used as a background. It follows that upon vacuum introduction, NO_2 leaves NO^+-NO_2 complex, restoring the original NO^+ band.

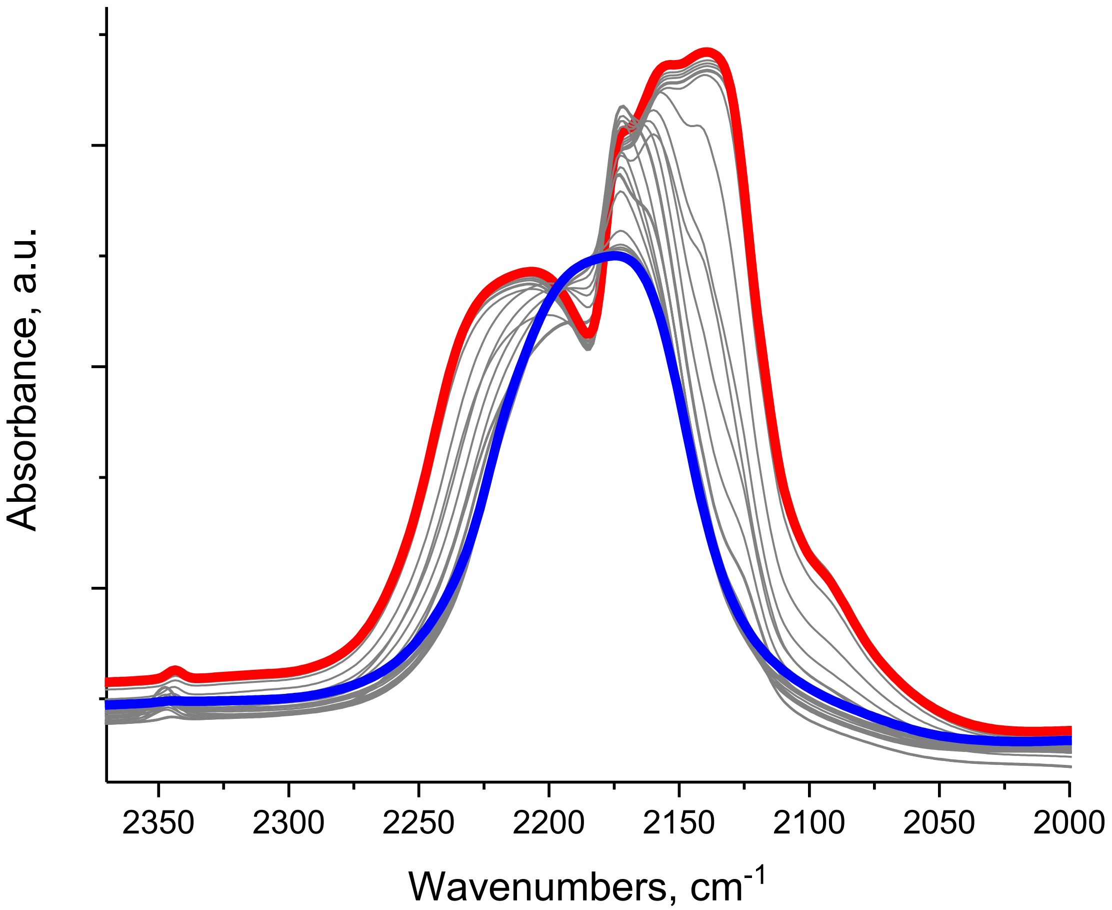

Supplementary Figure 9. In-situ FTIR during heating of NO^+-CO/H-SSZ-13 (Si/Al ~12). NO and sub-stoichiometric quantities of O_2 were first reacted with zeolite to produce NO^+ with only traces of nitrate, then vacuumed at 298 K, cooled down to 100 K, and reacted with 2 Torr CO at this temperature during continuous heating from 100 K (red spectrum) to 270 K (red spectrum). Only trace CO_2 evolution was observed. The intermediate (gray) spectra show evolution of the spectra during heating from 100 to 270 K. During this, no CO_2 evolves as evidenced by the lack of C-O stretches in the ~2345 cm^{-1} region. Thus, in the absence of nitrate no CO_2 evolution is observed.

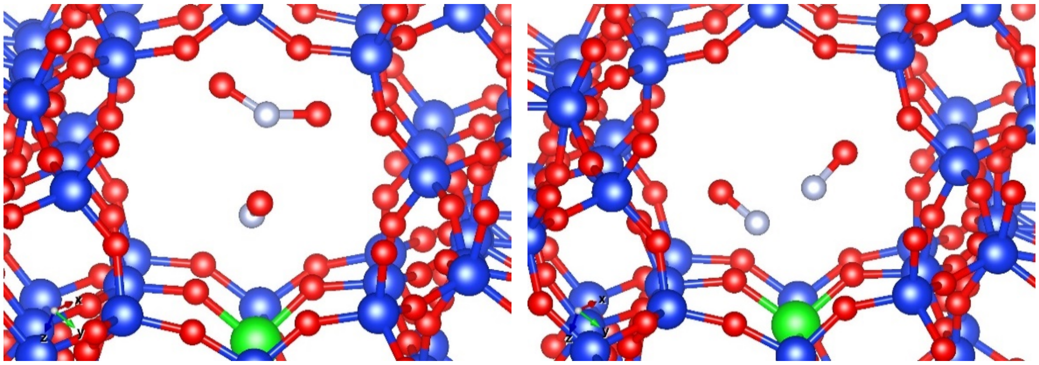

Supplementary Figure **10.** Structures of NO^+-NO_2/Zeolite (left panel) and NO^+-NO/Zeolite (right panel) complexes. Color coding: Si – blue, O – red, Al – green, N – light blue, C – brown, H – white.

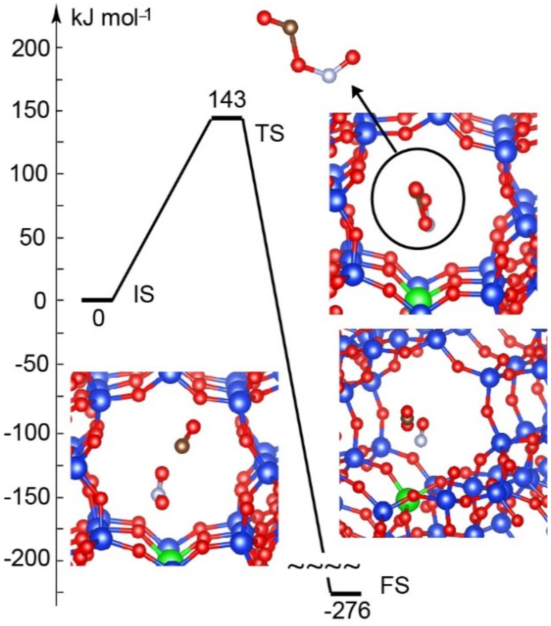

Supplementary Figure 11. Energy diagram of the mechanism of CO oxidation by NO_2^+-CHA. The corresponding initial state, transition state, and final state structures are also shown. Color coding: Si – blue, O – red, Al – green, N – light blue, C – brown, H – white.

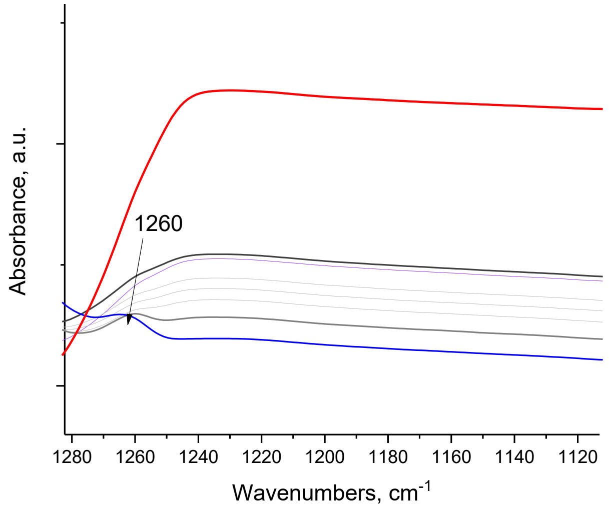

Supplementary Figure 12. In-situ FTIR of HON bending region during increase of temperature from 100 K (red line, described in Figure 2A, H-SSZ-13 with Si/Al ~12) to 135 K (blue line). The inset shows the magnified 2,400-2,000 cm^{-1} region. Zeolite pellet itself was used as IR background.

Supplementary Tables

Supplementary Table 1

Binding energy (in kJ/mol) of NO_2 and NO to Zeo/NO^+ structure, vibrational frequencies (in cm^{-1}), ν(N-O), as well as selected interatomic distances, R(A-B), in pm.

Structure	BE	ν(N-O)	R(N-N)	R(N-O)	R(O_{zeo}-N(NO^+)) [b]
NO^+ [a]		2404		109	
NOF [a]		1920		115	
ONNO [a]		1727, 1879	199		
Zeo/NO^+-NO_2	-21	1722, 2042	272	113.1	214, 222
Zeo/NO^+-NO	-52	2009, 1911	229	114.4, 114.6	223, 232

[a]gas phase species; [b]distances between zeolite O center and N atom from the charge-compensating NO^+ species

Supplementary Table 2

Binding energy (in kJ/mol) of NO_2 and NO to Zeo/NO^+ structure, as well as calculated (ν_{calc}) with different funcationals and experimental (ν_{exp}) vibrational frequencies (in cm^{-1}).

Structure	BE	ν_{calc}	BE	ν(N-O)	BE	ν_{calc}	ν_{exp}
	PBE+D2	PBE+D2	HSE06	HSE06	SCAN	SCAN	
Zeo/NO^+-NO_2	-21	1722, 2042	-19	1816, 2239	-37	2034, 2285	1746, 2080
Zeo/NO^+-NO	-52	1911, 2009	-21	2013, 2189	-52	2165, 2356	1870, 2013
CO (gas)		2130		2230		2294	2143

Supplementary Table 3

Relative energies with respect to the zeolite system at initial state (ZEO1) and CO and HNO_3 molecules in gas phase (E_{rel}), as well as with respect to the previous step (E_{diff}). Energy barriers ($E^{\#}$) are also shown. All energies (in kJ/mol) are calculated using PBE+D and HSE06 functionals.

	E_{rel} PBE+D	$E^{\#}$ PBE+D	E_{diff}` PBE+D	E_{rel} HSE06	$E^{\#}$ HSE06	E_{diff}` HSE06
**Mehanism A**						
ZEO1+CO(g)+HNO_3(g)	0			0		
ZEO1/CO/HNO_3	-116		-116	-117		-117
TS1	81	196		126	243	
ZEO1/CO_2/HNO_2	-332		-217	-332		-215
**Mehanism B**						
ZEO1+CO(g)+HNO_3(g)	0			0		
ZEO1/CO/HNO_3	-116		-116	-117		-117
TS2	-34	81		45	162	
ZEO1/CO_2/HNO_2	-332		-217	-332		-215
**Mehanism C**						
ZEO1+CO(g)+HNO_3(g)	0			0		
ZEO1/CO/HNO_3	-116		-116	-117		-117
TS2	-34	81		45	162	
ZEO2/NO_2/HCO_2	-105		10	-98		19
TS2	-34	71		45	143	

ZEO1/CO_2/HNO_2	-332		-227	-332		-234
**Mehanism D**						
ZEO1+CO(g)+HNO_3(g)	0			0		
ZEO1/CO/HNO_3	-116		-116	-117		-117
TS2	-34	81		45	162	
ZEO2/NO_2/HCO_2	-105		10	-98		19
ZEO2/HCO_2/NO_2	-116		-11	-96		2
TS3	-5	111		78	175	
ZEO2/CO_2/HNO_2	-300		-184	-298		-202
